# Supplementary material for: Pharmacokinetic Basis for Using Saliva Matrine Concentrations as a Clinical Compliance Monitoring in Antitumor B Chemoprevention Trials in Humans
Source: Cancers (Basel). 2022 Dec 23;15(1):89. doi: 10.3390/cancers15010089 (PMC9817974; doi:10.3390/cancers15010089)
Supplement: Supplementary file 1 [file cancers-15-00089-s001.zip › S1 Quantitive Analysis.pdf]

Supplementary materials for

Pharmacokinetic Basis for Using Saliva Matrine Concentrations as a Clinical Compliance Monitoring in Antitumor B Chemoprevention Trials in Humans

Dinh Bui[1], Lenora A. McWilliams[2*], Lei Wu[1*], Haiying Zhou[3], Stuart Wong[4], Ming You[4,5], Diana S-L. Chow[1], Rashim Singh[1], Ming Hu[1]

[1] Department of Pharmacological and Pharmaceutical Sciences, College of Pharmacy, The University of Houston, Houston, Texas

[2] College of Nursing, The University of Houston, Sugar Land, Texas

[3] Simulations Plus, Inc. Lancaster, California

[4] Department of Pharmacology and Toxicology, Medical College of Wisconsin, Milwaukee, Wisconsin

[5] Current Address: Center for Cancer Prevention, Houston Methodist Cancer Center, Houston, Texas

(*): These co-authors are equal contribution to the paper

Corresponding authors

[Tel: (832) 842-8320; Fax: (713) 743-1884; E-mail: mhu@uh.edu]

[Tel: (832) 518-9110; E-mail: rsingh3@Central.uh.edu]

S1. Quantitative Analysis of Stability and Human PK Samples

S1.1 UPLC Method Development

UPLC conditions for analyzing ATB key active components were: system, Waters Acquity™; column, BEH C18 column (50 × 2.1 mm I.D., 1.7 μm, Waters, Milford, MA, USA); mobile phase A (MPA), 2 mM ammonium acetate in water; mobile phase B (MPB), 100 % acetonitrile; gradient, 0 – 0.5 min, 5 % MPB, 0.5 –1.5 min, 5 – 30 % MPB, 1.5 – 4.0 min, 30 – 60 % MPB, 4.0 – 5.0 min, 60 – 80 % MPB, 5.0 – 5.5 min, 80 - 95 % MPB, 5.5 – 6.0 min, 95 % MPB, 6.0 - 6.1 min, 95 - 5 % MPB, 6.1 – 6.5 min, 95 % MPB; flow rate, 0.45 mL/min; column temperature, 45 °C; injection volume, 10 μL.

S1.2 UPLC-MS/MS Method Development and Validation

The method used to analyze the study samples was developed and validated based on FDA's Bioanalytical Method Validation Guidance for Industry (2018 version).

Concentrations of ATB KACs in biological matrices were determined by using MRM (Multiple Reaction Monitoring) method in negative and positive mode simultaneously. The instrument dependent parameters for mass spectrum were set as following: ionspray voltage, -4.5 kV (negative mode), 5500 kV (positive mode); ion source temperature, 600 °C; nebulizer gas (gas 1), nitrogen, 50 psi; turbo gas (gas 2), nitrogen 50 psi; curtain gas, nitrogen 20 psi. Unit mass resolution was set in both mass-resolving quadruples Q1 and Q3.

S1.2.1 Method optimization

The method was established by optimizing UPLC and MS/MS condition to obtain the best possible sensitivity. Methanol, acetonitrile, 2 mM ammonium acetate (pH = 7.6), 0.1 % formic acid (pH = 2.5), and 100% water were tested as potential mobile phases. The ionization of Matr, Dict, Maac and Frax in the instrument was the best with acetonitrile and 2 mM ammonium acetate

as the mobile phases. A gradient elution was used to avoid cross peaks. To obtain a sharp and symmetrical peak, the column temperature was set up at 45ºC and the flow rate was 0.45 mL/min. Under this optimized elution scheme, a specific MRM scan was used to improve the analysis specificity. The compounds and instrument dependent parameters were optimized by tuning these analytes in positive and negative scan mode and their results are shown in **Table S1**. Baohuoside I was used as an internal standard (I.S.) in this method.

S1.2.2 Sample Processing

Standard Samples. Calibration standard samples were prepared in 50% MeOH by diluting stock solutions to the working standard solutions of 10000.0, 5000.0, 2500.0, 1250.0, 625.0, 312.5, 156.3, 78.1, 39.1, 19.5, 9.8, 4.9 nM, respectively for Matr, Maac, and Frax, whereas 1000.0, 500.0, 125.0, 62.5, 31.3, 15.6, 7.8, 3.9, 2.0, 1.0, 0.5 nM for Dict. The calibration standard samples were prepared by spiking 10 μL of blank plasma with 10 μL of the above working solutions and 180 μL of I.S. in EtOAc : MeOH 4 : 1.

PK Samples. The plasma or saliva sample (20 μL) was spiked with 20 μL of 50% MeOH and 160 μL of 100 nM I.S. in ethyl acetate. The mixture was vortexed for 1 min. After centrifugation at 17,968 x g for 15 min, the supernatant was transferred to a new microcentrifuge tube and evaporated to dryness under a stream of airflow. The residue was reconstituted in 140 μL of 50% methanol and centrifuged at 17,968 x g for 15 min. The supernatant (10 μL) was injected into the UPLC-MS/MS system for quantitative analysis. The samples were concentrated by lyophilizing large sample amounts (500 μL for plasma and 1000 μL saliva) and then extracted with the sample processing method of ATB to quantify the low concentration KACs.

Quality Control Samples. The quality control (QC) samples for each compound were prepared at three different concentrations (high, medium, and low) in the same way as the plasma/saliva

samples for calibration were prepared. The study and QC samples were prepared on the same day as the UPLC-MS/MS analysis was done.

S1.2.3 Method Validation

Calibration curves were prepared the same way as described in section S1.2.2. The linearity of each calibration curve was determined by plotting the peak area ratio of Matr, Maac, Dict, and Frax to I.S. in plasma. Least-squares linear regression method ($1/x^2$ weight) was used to determine the slope, intercept, and correlation coefficient of linear regression equation. The lower limit of detection (LLOD) was defined based on a signal-to-noise ratio of 10:1.

S1.2.4 Extraction recovery and matrix effect

The extraction recoveries of Matr, Maac, Dict, and Frax were determined by comparing the relative peak areas obtained from blank plasma spiked with analytes and those obtained from water spiked with the same amounts of analytes. Matrix effect was determined by comparing the peak areas of blank plasma extracts spikes with analytes and I.S. with those of the standard solutions dried and reconstituted with mobile phase. The quality control samples were prepared in plasma. Extraction recovery and matrix effect results are shown in **Table S3.**

S1.2.5 Sample Stability

Bench work (25°C for 4 hours), short-term (-20°C for 7 days), long-term (-80°C for 4 months) and three freeze-thaw cycle stabilities were determined. Results are shown in **Table S4.**

Table S1. Compound-dependent parameters of ATB compounds and internal standard Baohuoside I in UPLC-MS/MS analysis

Compound	Q1 (*m/z*)	Q3 (*m/z*)	DP(V)	CE(V)	CXP(V)	Dwell time (ms)
**Bao** (positive mode)	515.2	313.1	110	45	22	10
**Bao** (negative mode)	513.3	366.0	-8	-34	-14	20
**Maac**	283.2	254.1	- 166	- 24	- 14	20
**Matr**	249	148	189	39	9	10
**Dict**	200.4	185	120	35	13	10
**Frax**	233.4	129	90	46	20	10

Table S2. Recovery and matrix effect of Matr, Maac, Dict and Frax at high, medium, and low concentrations in human plasma and saliva

**Conc. level**		**Matr**	**Dict**	**Maac**	**Frax**
**LLOQ**	Recovery (%)	110.0 ± 3.3	84.9 ± 1.5	107.5 ± 12.8	95.2 ± 9.3
	Matrix effect (%)	114.7 ± 6.3	88.3 ± 4.0	111.4 ± 24.0	102.7 ± 8.9
**MQC**	Recovery (%)	89.5 ± 6.1	89.6 ± 3.3	82.6 ± 7.7	103.6 ± 2.5
	Matrix effect (%)	87.6 ± 1.5	94.6 ± 6.4	102.4 ± 4.8	85.7 ± 1.0
**HQC**	Recovery (%)	90.4 ± 12.7	96.0 ± 7.4	87.1 ± 12.6	101.4 ± 4.8
	Matrix effect (%)	88.1 ± 6.1	89.7 ± 2.8	107.3 ± 12.8	102.5 ± 6.6

Table S3. Stability results of ATB components in different conditions

Analytes	Conc.	**Bench work** (4 hrs, 25 ºC)	**3 cycles freeze-thaw**	**Short-term** (7 days, -20 ºC)	**Long-term** (6 months, -80 ºC)
**Matr**	LOQ	110.5 ± 18.0	109.6 ± 1.3	86.6 ± 3.8	96.2 ± 0.6
	MQC	85.8 ± 8.9	93.7 ± 3.4	85.7 ± 8.7	105.8 ± 3.8
	HQC	103.1 ± 2.6	99.1 ± 6.7	88.3 ± 6.6	94.6 ± 6.7
**Maac**	LOQ	93.8 ± 11.6	113.3 ± 6.4	99.6 ± 1.4	87.0 ± 5.1
	MQC	109.1 ± 5.9	114.6 ± 3.2	89.1 ± 11.0	85.5 ± 0.4
	HQC	108.7 ± 8.1	114.7 ± 2.8	91.2 ± 8.0	92.3 ± 5.7
**Dict**	LOQ	106.7 ± 1.9	110.2 ± 3.6	97.7 ± 13.6	85.1 ± 5.4
	MQC	97.3 ± 3.7	94.7 ± 7.6	85.4 ± 0.7	91.7 ± 5.4
	HQC	95.3 ± 13.2	101.9 ± 1.5	112.5 ± 7.2	113.1 ± 6.2
**Frax**	LOQ	108.5 ± 5.0	105.7 ± 3.2	89.6 ± 0.5	85.7 ± 3.6
	MQC	90.5 ± 8.0	104.0 ± 1.3	96.6 ± 5.8	90.1 ± 5.6
	HQC	89.1 ± 9.9	98.9 ± 2.2	99.1 ± 8.0	94.8 ± 0.4

Table S4. Stability results of ATB tablets represented as remaining percentage of ATB KACs after storage in different conditions

**Storage Condition**	**Accelerated term**		**Intermediate**		**Long term**		
	3 months	6 months	3 months	6 months	3 months	6 months	12 months
Maac	110.5 ± 18.0	109.6 ± 1.3	86.6 ± 3.8	96.2 ± 0.6	81.1 ± 5.3	101.6±0.2	89.1 ± 4.2
Dict	103.8 ± 9.9	108.3 ± 3.2	119.1 ± 11.0	126.2 ± 0.4	107.1 ± 6.8	93.4 ± 3.7	97.7 ± 6.2
Frax	84.3 ± 13.2	115.8 ± 1.5	112.5 ± 7.2	113.1 ± 7.2	92.4 ± 6.4	81.2 ± 10.1	93.8 ± 1.0
Matr	87.0 ± 5.0	99.4 ± 3.2	89.6 ± 0.5	92.1 ± 3.6	89.1 ± 2.7	93.9 ± 2.1	89.6 ± 0.8
