# Supplementary material for: Pharmacokinetic Basis for Using Saliva Matrine Concentrations as a Clinical Compliance Monitoring in Antitumor B Chemoprevention Trials in Humans
Source: Cancers (Basel). 2022 Dec 23;15(1):89. doi: 10.3390/cancers15010089 (PMC9817974; doi:10.3390/cancers15010089)
Supplement: Supplementary file 1 [file cancers-15-00089-s001.zip › S2_Human Pharmacokinetic Study Design_Study participant.pdf]

Supplementary materials for

Pharmacokinetic Basis for Using Saliva Matrine Concentrations as a Clinical Compliance Monitoring in Antitumor B Chemoprevention Trials in Humans

Dinh Bui[1], Lenora A. McWilliams[2*], Lei Wu[1*], Haiying Zhou[3], Stuart Wong[4], Ming You[4,5], Diana S-L. Chow[1], Rashim Singh[1], Ming Hu[1]

[1] Department of Pharmacological and Pharmaceutical Sciences, College of Pharmacy, The University of Houston, Houston, Texas

[2] College of Nursing, The University of Houston, Sugar Land, Texas

[3] Simulations Plus, Inc. Lancaster, California

[4] Department of Pharmacology and Toxicology, Medical College of Wisconsin, Milwaukee, Wisconsin

[5] Current Address: Center for Cancer Prevention, Houston Methodist Cancer Center, Houston, Texas

(*): These co-authors are equal contribution to the paper

S2.1. Human Pharmacokinetic Study Design

The study was conducted in two phases. Two self-volunteered subjects participated in the study on Jan 14th, 2020 whereas rest of the 6 subjects participated on Jan 21st, 2020. Participant fasted for 8-12 hours before the administration of ATB. Participants refrained from consumption of caffeinated drinks starting the night before the study and ending after the 24-hour sample. A saline lock was inserted in the arm of each participant at the start of the study in the morning and pre-dosing blood and saliva samples were collected. Each participant received single-dose oral administration of 8 tablets of ATB (300 mg ATB per tablet) with 250 ml water. Blood (approximately 2 ml) and saliva (approximately 2 ml) samples were collected by spitting into the Salimetric collection kit at pre-determined time points (0.5, 1, 2, 3, 4, 6, 8, and 24 hours). Volunteers were asked to stop drinking or eating 15 minutes before sampling and spit into the saliva sample kit for salivary collection. Plasma samples were then collected by centrifugation of blood samples at 4 °C, 3,200 g for 15 minutes. Breakfast was provided at 30-min and lunch at 3-hour post-administration. Water and juice were permitted as desired except within 30 mins of administration. Participants avoided any drink 15 mins before each saliva sample. Throughout the study, the participants waited in a pre-assigned area with the nurses till the 8-hour samples were collected and saline lock was removed. Participants returned the next day morning for 24-hour sample collection. Blood samples were also collected for biochemistry labs before enrollment in the study and 24-hours after the administration of ATB. Participants fasted 8-12 hours before each blood draw for labs. Safety was assessed by observing the participants during the study, participant-reported adverse events next day, and blood biochemistry laboratory assessments.

S2.2. Study Participants

Eligible participants were generally healthy men and women with no diagnosed disease or illness, more than 18 and less than 40 years old were enrolled. Key exclusion criteria were history of active liver disease or cancer; severe current or recurrent comorbidity such as (e.g., cardiovascular, hematological, neurological, endocrine, renal, liver, GI, HIV-AIDS, or other conditions such as cancer) that could affect the absorption and/or disposition of study drug; any disease/illness diagnosed by a licensed physician; positive for HIV and/or Hepatitis B and C tests; has had an acute illness within two weeks prior to screening; concurrent use of any prescription medication (including medicinal botanical) except birth control pills, over the counter medication and supplements except Vitamins and mineral supplements, or herbal supplements in form of herbal mixtures, teas or individual compounds (such as quercetin, curcumin, echinacea, flaxseed, ginseng, ginkgo, soy etc.) that could potentially impact the results/objectives of this study; concurrent use of recreational drugs or alcohol during the study (self-declared by study participants); any clinically important abnormalities in blood chemistry (including alanine aminotransferase (ALT), aspartate aminotransferase (AST), alkaline phosphatase (ALP), gamma-glutamyl transferase (GGT), total bilirubin or serum creatinine levels above the upper limit of normal). Pregnant or lactating women were excluded due to unforeseeable risks to embryo or fetus. Prisoners and economically and/or educationally disadvantaged persons were excluded.

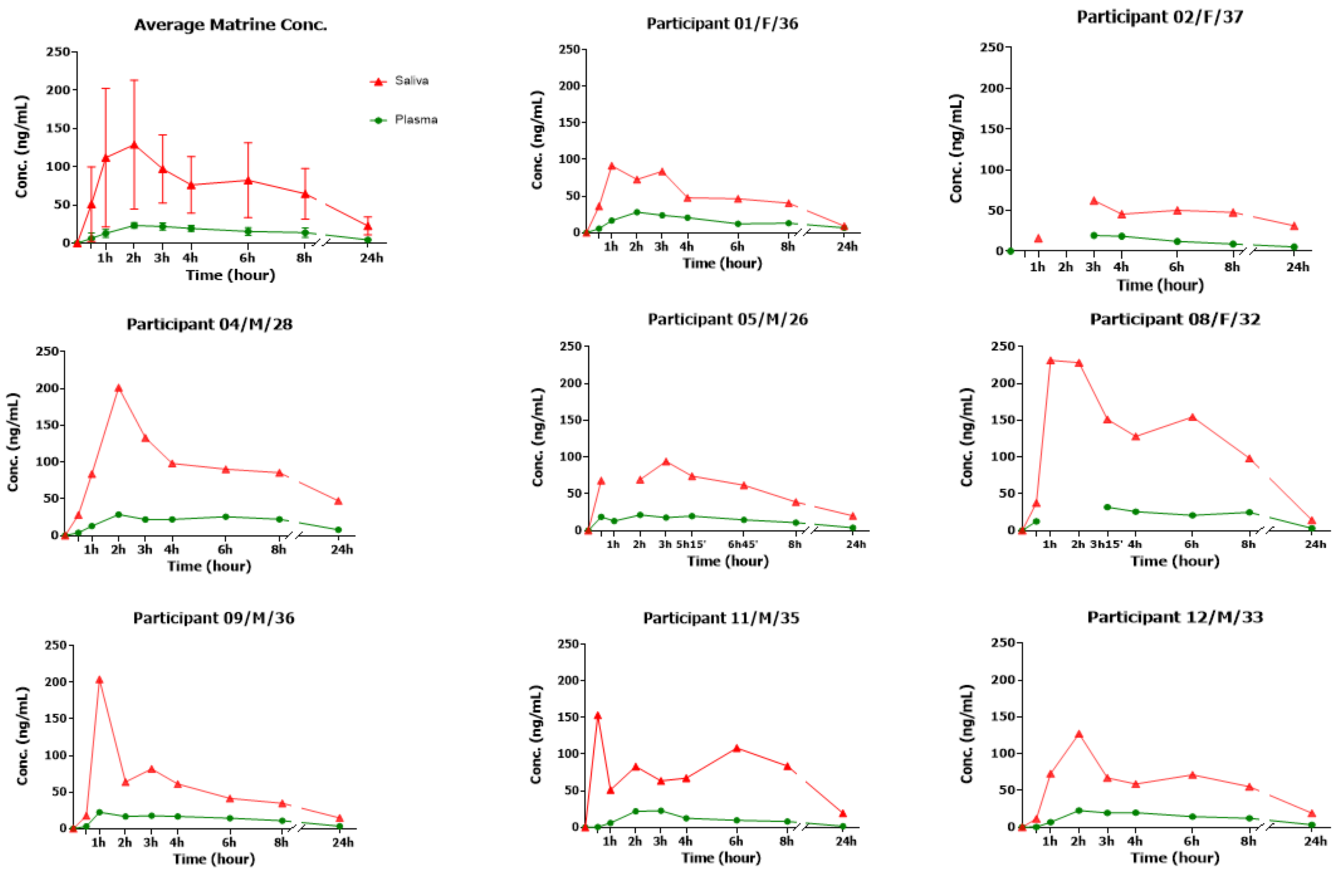

Figure S1. Average and detailed pharmacokinetic profile of observed matrine concentrations in plasma and saliva of individual participants

Table S5. List of blood tests performed for enrollment screening and 24 hrs after Antitumor B (ATB) dosing

**Screening Tests**	**ATB Post-dosing Tests**
Fasting Glucose	Fasting Glucose
BUN	BUN
Creatinine	Creatinine
eGFR Afraican American	eGFR Afraican American
eGFR Non-afraican American	eGFR Non-afraican American
Calculated BUN/Creatinine	Calculated BUN/Creatinine
Sodium	Sodium
Potassium	Potassium
Chloride	Chloride
Carbon dioxide	Carbon dioxide
Calcium	Calcium
Protein, Total	Protein, Total
Albumin	Albumin
Calculated Globulin	Calculated Globulin
Calculated A/G Ration	Calculated A/G Ration
Bilirubin, Total	Bilirubin, Total
Alkaline Phosphatase	Alkaline Phosphatase
AST	AST
ALT	ALT
WBC	WBC
RBC	RBC
Hemoglobin	Hemoglobin
Hematocrit	Hematocrit
MCV	MCV
MCH	MCH
MCHC	MCHC
RDW	RDW
Neutrophils	Neutrophils
Lympocytes	Lympocytes
Monocytes	Monocytes
Eosinophils	Eosinophils
Basophils	Basophils
Platelet Count	Platelet Count
GGT	GGT
Magnesium	Magnesium
Hepatitis B Surface AG	
Hepatitis C Antibody	
HIV 1/2 4th Gen, RFLX CONF	

Table S6. Summary of Participants' Demographic, Post-study Feedback, and Blood Biochemistry Changes 24 hours after ATB dosing

							Changes in Blood Biochemistry					
**Participant ID**	**Sex**	**Race**	**Age**	**Weight (lbs)**	**Height (cm)**	**BMI**	**Test**	**Before**	**After**	**Reference range**	**Observation of the subject during study**	**Participant feedback 24 hr after the study**
01/F/36	F	Caucasian	36	114	158.5	20.6	Creatinine	0.53*	0.48*	0.80-1.40 mg/dL	Normal	No complaints
							WBC	3.8*	6.3	4.0-11.0 K/UL		
							Hemoglobin	11.2*	11.5*	13.0-17.0 G/dL		
02/F/37	F	Asian	37	192.2	165	32	MCHC	32.5	31.7*	32.0-35.5 G/dL	Difficulty in blood draw	No complaints
04/M/28	M	Asian	28	146.2	167	23.8	Creatinine	0.7*	0.76*	0.80-1.40 mg/dL	Normal	Feeling very sleepy during and after 8 hr sample
05/M/26	M	Asian	26	145	176	21.2	BUN	11*	24	6-20 mg/dL	Difficulty in blood draw	Feeling very tired after 8 hr blood sample
							Bilirubin, Total	1.3*	0.7	<=1.2 mg/dL		
08/F/32	F	Asian	32	103	151	20.5	No Change				Difficulty in blood draw	No complaints
09/M/36	M	Caucasian	36	190.8	171	29.6	MCHC	35.6*	34.8	32.0-35.5 G/dL	Normal	No complaints
11/M/35	M	Asian	35	169.2	170	26.6	RBC	5.85*	5.72*	4.10-5.70 M/UL	Normal	No complaints
							Hemoglobin	17.9*	17.6*	13.0-17.0 G/dL		
							Hematocrit	50.4*	49.9*	37.0-49.0%		
12/M/33	M	Asian	33	150.8	159	27.1	Creatinine	0.85	0.79*	0.80-1.40 mg/dL	Nausea and vomiting before saline lock was inserted, normal during the study	No complaints
							WBC	4.5	3.8*	4.0-11.0 K/UL		

*** stands for out of normal range**
