# Supplementary material for: Pharmacokinetic Basis for Using Saliva Matrine Concentrations as a Clinical Compliance Monitoring in Antitumor B Chemoprevention Trials in Humans
Source: Cancers (Basel). 2022 Dec 23;15(1):89. doi: 10.3390/cancers15010089 (PMC9817974; doi:10.3390/cancers15010089)
Supplement: Supplementary file 1 [file cancers-15-00089-s001.zip › S3_Compartmental PK Co-modeling.pdf]

Supplementary materials for

Pharmacokinetic Basis for Using Saliva Matrine Concentrations as a Clinical Compliance Monitoring in Antitumor B Chemoprevention Trials in Humans

Dinh Bui[1], Lenora A. McWilliams[2*], Lei Wu[1*], Haiying Zhou[3], Stuart Wong[4], Ming You[4,5], Diana S-L. Chow[1], Rashim Singh[1], Ming Hu[1]

[1] Department of Pharmacological and Pharmaceutical Sciences, College of Pharmacy, The University of Houston, Houston, Texas

[2] College of Nursing, The University of Houston, Sugar Land, Texas

[3] Simulations Plus, Inc. Lancaster, California

[4] Department of Pharmacology and Toxicology, Medical College of Wisconsin, Milwaukee, Wisconsin

[5] Current Address: Center for Cancer Prevention, Houston Methodist Cancer Center, Houston, Texas

(*): These co-authors are equal contribution to the paper

S3. Compartmental Pharmacokinetic Co-modeling

The population PK model was developed using plasma and saliva concentrations of 8 healthy participants in the study. Although there were some missing data points due to technical difficulty in blood sampling, no input was made for the missing data points. Initial estimates of individual compartmental PK parameters were derived using WinNonlin® version 8.2. The concentration of matrine in plasma and saliva were fitted simultaneously. Actual dosing and sampling times were used for the compartmental modeling. Different compartmental PK models were tested to describe the concentration of matrine in plasma and saliva compartments (**Fig 7** in the main manuscript). The final model (model C) was described by the mass balance equations listed below:

$$A1 = - (Cl\_p * C) + (Aa * Ka) - (Vmax * C / (C + Km)) - (A1 * K13) + (A2 * Ksp)$$

$$Aa = - (Aa * Ka) - (Aa * K\_a2)$$

$$A2 = (Vmax * C / (C + Km)) - (CL\_s * Cs) + (A3 * Kts) - (A2 * Ksp) + (Aa * K\_a2)$$

$$A3 = (A1 * Kpt) - (A3 * Kts)$$

The population pharmacokinetic compartmental models using actual dosing and sampling times were built using Phoenix NLME 8.2. To determine the best fit pharmacokinetic model structure, model selection and identification of variability were based on minimizing the Akaike Information Criteria (AIC) and the log-likelihood values (-2LL), achieving adequate parameter precision, inspection of goodness-of-fit plots and by comparison of the quality of fit plots (such as observed vs. fitted data, weighted residual vs. fitted data, weighted residual vs. time).

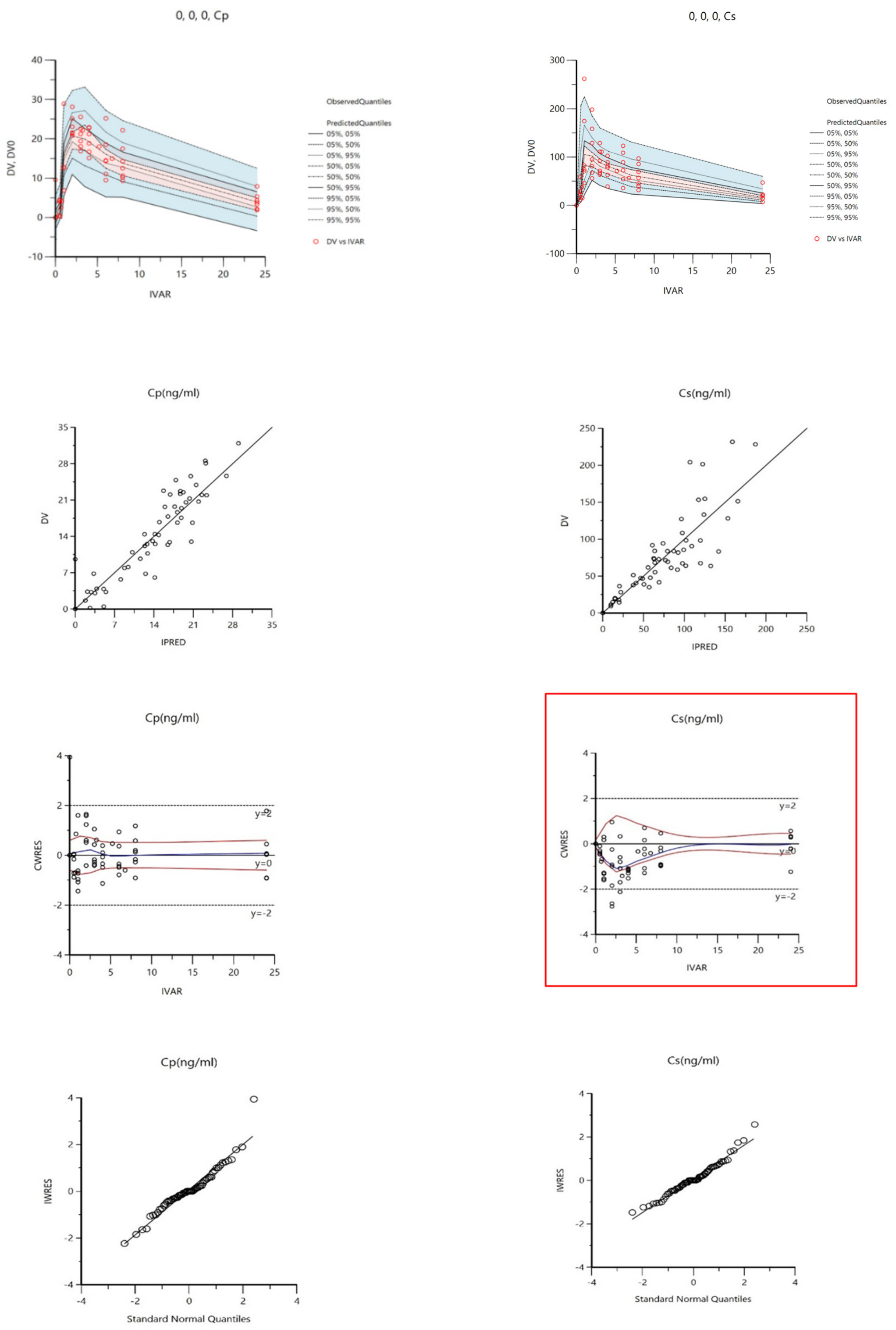

Figure S2. Best fit model result plots of matrine in human plasma and saliva (model B)

A B

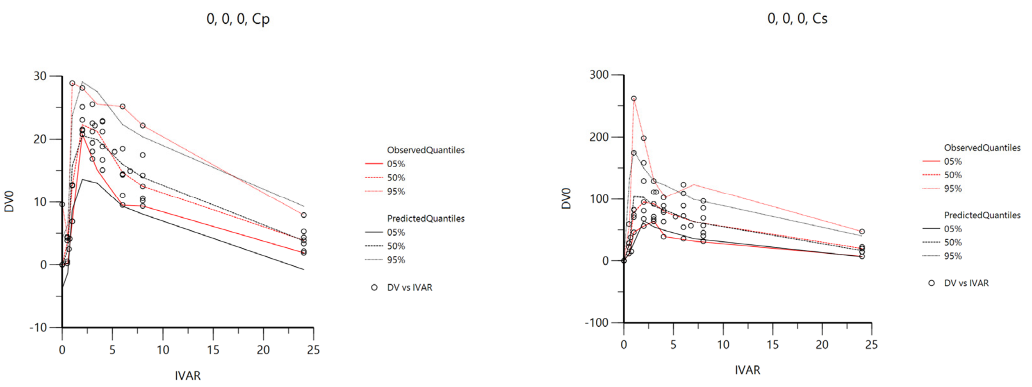

Figure S3. Visual Prediction Check Plots show the observed and predicted quantiles at 5, 50, and 95% levels of the plasma concentration (A) and saliva concentration (B)
