# Supplementary material for: Pharmacokinetic Basis for Using Saliva Matrine Concentrations as a Clinical Compliance Monitoring in Antitumor B Chemoprevention Trials in Humans
Source: Cancers (Basel). 2022 Dec 23;15(1):89. doi: 10.3390/cancers15010089 (PMC9817974; doi:10.3390/cancers15010089)
Supplement: Supplementary file 1 [file cancers-15-00089-s001.zip › S4_PBPK Modeling and Interspecies Scaling.pdf]

Supplementary materials for

Pharmacokinetic Basis for Using Saliva Matrine Concentrations as a Clinical Compliance Monitoring in Antitumor B Chemoprevention Trials in Humans

Dinh Bui[1], Lenora A. McWilliams[2*], Lei Wu[1*], Haiying Zhou[3], Stuart Wong[4], Ming You[4,5], Diana S-L. Chow[1], Rashim Singh[1], Ming Hu[1]

[1] Department of Pharmacological and Pharmaceutical Sciences, College of Pharmacy, The University of Houston, Houston, Texas

[2] College of Nursing, The University of Houston, Sugar Land, Texas

[3] Simulations Plus, Inc. Lancaster, California

[4] Department of Pharmacology and Toxicology, Medical College of Wisconsin, Milwaukee, Wisconsin

[5] Current Address: Center for Cancer Prevention, Houston Methodist Cancer Center, Houston, Texas

(*): These co-authors are equal contribution to the paper

S4. PBPK modeling and interspecies scaling

The PBPK model of matrine in blood/plasma was developed using GastroPlus 9.8® software (Simulations Plus, Inc., Lancaster, CA). All physiological parameters have parameterized *a priory* in the software. The blood/plasma ratio, solubility, permeability parameters of matrine were used as reported. [1], [2] The LogD, pKa parameters were used as predicted by the ADMET Predictor™ 9.5 (Simulations Plus). Due to the significance of the first pass effect in the gut as reported in the mice studies, the gut metabolism of matrine was added to the model.[3] The clearance mechanism of Matr was described in the PBPK model by the first pass metabolism in the intestine and the active secretion in the kidney [4]. The OCT2 transporter was included in the model because it is expressed in the kidney and salivary gland.[5], [6] The parameters used to develop the PBPK model of matrine were listed in **Table 6** in the main manuscript.

The PBPK model was evaluated by comparing model simulated results with the experimental data in rodents and humans after oral administration of ATB. The PBPK model was verified if model simulation described the experimental data (C_{max}, T_{max}, AUC) well. The model was also validated externally by simulating the plasma concentration with the dose of 4.8 mg matrine in American males and compare with the reported data by Gao *et al*.[9]

The PBPK model was scaled up to humans by substituting the species-dependent physiological parameters, pharmacokinetic parameters such as clearances, and partition coefficients of the rodent models with human specific values. The Kp value were calculated using the Lukacova method (Gastro Plus 9.8). The optimized parameter values were listed in **Table 6** in the main manuscript. Observed data for model development were determined using a single-dose oral PK of ATB suspension 500 mg/kg (in mice)/250 mg/kg (in rats) and 8 tablets (300 mg of ATB/tablet) in humans. The equivalent dosages in rodents were calculated from the human dose according to the guidance of Nair *et al* [10].

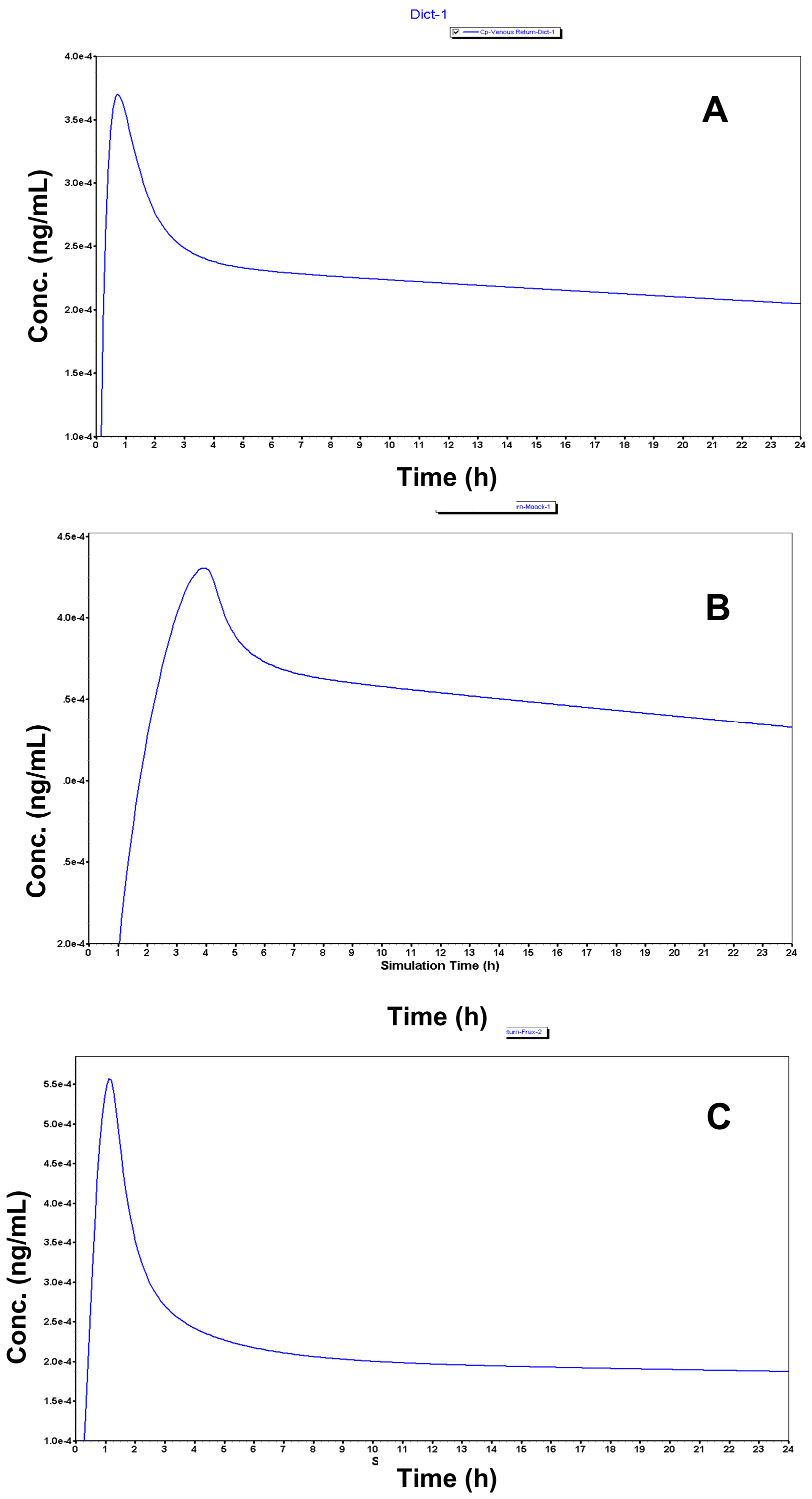

Figure S3. Predicted plasma PK profiles of dictamnine (A), maackiain (B), and fraxinellone (C) in healthy adults after single dose oral administration of 8 ATB tablets with GastroPlus

There was significant difference in the bioavailability of Matrine in rats (17.1 ± 5.4%) [8] as compared to mice (2.4 ± 1.2%) [3]. Physiological conditions such as pH, bile, pancreatic juice, and mucus and fluid volume and content in the gastrointestinal tract can modify dissolution rates, solubility, transit times, and membrane transport, metabolism enzymes, and enterohepatic recycling of drug molecules. This difference could be attributed either to difference in intestinal absorption among species or to the low clearance of Matr in rats. Those values were therefore optimized for best fit with the observed data for each species.

References

[1] G. Gao, "Commparative pharmacokinetics of matrine: pure matrine vs. crude chemical in ACAPHA," Simon Fraser University, 2007.

[2] G. Gao and F. C. P. Law, "Physiologically based pharmacokinetics of matrine in the rat after oral administration of pure chemical and ACAPHA," *Drug Metab. Dispos.*, vol. 37, no. 4, pp. 884–891, 2009.

[3] D. Bui *et al.*, "Pharmacokinetic Characterization and Bioavailability Barrier for the Key Active Components of Botanical Drug Antitumor B (ATB) in Mice for Chemoprevention of Oral Cancer," *J. Nat. Prod.*, Aug. 2021.

[4] G. Gao and F. C. P. Law, "Physiologically based pharmacokinetics of matrine in the rat after oral administration of pure chemical and ACAPHA," *Drug Metab. Dispos.*, vol. 37, no. 4, pp. 884–891, 2009.

[5] N. Lee, H. Duan, M. F. Hebert, C. J. Liang, K. M. Rice, and J. Wang, "Taste of a pill:

Organic cation transporter-3 (OCT3) mediates metformin accumulation and secretion in salivary glands," *J. Biol. Chem.*, vol. 289, no. 39, pp. 27055–27064, 2014.

[6] H. Motohashi and K. I. Inui, "Organic cation transporter OCTs (SLC22) and MATEs (SLC47) in the human kidney," *AAPS Journal*, vol. 15, no. 2. pp. 581–588, 2013.

[7] A. Nair and S. Jacob, "A simple practice guide for dose conversion between animals and human," *J. Basic Clin. Pharm.*, 2016.

[8] Z. Yang *et al.*, "Biopharmaceutical and pharmacokinetic characterization of matrine as determined by a sensitive and robust UPLC-MS/MS method," *J. Pharm. Biomed. Anal.*, 2010.
