# Supplementary material for: Pharmacokinetic Basis for Using Saliva Matrine Concentrations as a Clinical Compliance Monitoring in Antitumor B Chemoprevention Trials in Humans
Source: Cancers (Basel). 2022 Dec 23;15(1):89. doi: 10.3390/cancers15010089 (PMC9817974; doi:10.3390/cancers15010089)
Supplement: Supplementary file 1 [file cancers-15-00089-s001.zip › S5_Recyling of Matrine.pdf]

Supplementary materials for

Pharmacokinetic Basis for Using Saliva Matrine Concentrations as a Clinical Compliance Monitoring in Antitumor B Chemoprevention Trials in Humans

Dinh Bui[1], Lenora A. McWilliams[2*], Lei Wu[1*], Haiying Zhou[3], Stuart Wong[4], Ming You[4,5], Diana S-L. Chow[1], Rashim Singh[1], Ming Hu[1]

[1] Department of Pharmacological and Pharmaceutical Sciences, College of Pharmacy, The University of Houston, Houston, Texas

[2] College of Nursing, The University of Houston, Sugar Land, Texas

[3] Simulations Plus, Inc. Lancaster, California

[4] Department of Pharmacology and Toxicology, Medical College of Wisconsin, Milwaukee, Wisconsin

[5] Current Address: Center for Cancer Prevention, Houston Methodist Cancer Center, Houston, Texas

(*): These co-authors are equal contribution to the paper

S5. Recycling of Matrine

The PK of matrine in oral ATB can be explained by its salivary excretion followed by the unique recycling phenomena, in which matrine excreted into saliva is absorbed back into the systemic circulation either by the oral epithelium (oral-saliva recycling) or to a larger extent by the enterocytes (entero-saliva recycling) (**Fig 4** in the main manuscript). The entero-saliva recycling pathway is responsible for the extended elimination phase in its plasma PK profile and increased $T_{1/2}$ and plasma AUC of matrine. As a result, the drug half-life values were long ($t_{1/2}$ matrine = 13.4 ± 6.9 hours in saliva and 10.0 ± 2.8 hours in plasma). With the daily saliva secretion volume of 500 – 600 mL per day in adult human,[11] a significant quantity of matrine and other drugs (e.g., metformin, amphetamine, theophylline, isoniazid, phenobarbital, phenytoin, lithium, acetaminophen) excreted in saliva are expected to enter the oral-saliva and entero-saliva recycling. The intrinsic clearance value of matrine in mice was found to be significantly higher than the reported number in rats and human [12]. The different may come from the very high concentration of matrine found in the mice salivary glands and higher ratio of the salivary glands size compared to the body weight in mice than rats and humans.
